# The evolutionary origin of human kissing

**DOI:** 10.1002/evan.22050

**Published:** 2024-10-17

**Authors:** Adriano R. Lameira

**Affiliations:** ^1^ ApeTank, Department of Psychology University of Warwick Coventry UK

**Keywords:** affection, ancestral ape, great apes, grooming, human evolution, kissing, social ties, “groomer's final kiss hypothesis”

## Abstract

A kiss has been a signal of special affection across continents and cultures for millennia. Between times and peoples, social norms invariably prescribe kissing to specific affiliations and contexts, implying deeper biological bases. Why the protruding of the lips and slight suction when touching another? Capuchin monkeys stick their fingers in their friends' eyes as sign of affection, why have humans developed kissing? Here I briefly review proposed hypotheses for the evolution of human kissing. Great ape social behavior suggests that kissing is likely the conserved final mouth‐contact stage of a grooming bout when the groomer sucks with protruded lips the fur or skin of the groomed to latch on debris or a parasite. The hygienic relevance of grooming decreased over human evolution due to fur‐loss, but shorter sessions would have predictably retained a final “kissing” stage, ultimately, remaining the only vestige of a once ritualistic behavior for signaling and strengthening social and kinship ties in an ancestral ape.

## 1 KISSING IN PLEASANTVILLE

Few natural human signals carry the symbolism and social sanctions of kissing. Cultural conventions across history and civilizations have dictated for (at least) 4500 years how close affection can be publicly shown and privately demonstrated, who can kiss, where one can kiss, when, how, and how many times.^1^ For example, Romans had several types of kisses that played different roles: the osculum (a kiss on the cheek signifying social or familiar affection without romantic implications, also used as a sign of politeness), the basium (a kiss on the lips signifying closer relationship between close family members or lovers but without sexual meaning), and the savium (a kiss on the lips signifying erotic or sexual desire between romantic partners).

Today, in Latin Europe, two left‐right cheek‐to‐cheek kisses may be used as greeting between woman and cross‐sex, not between men (they shake hands), unless they are of close kinship or during special ceremonies (e.g., funeral), but strong differences exist between regions, social strata, and/or contexts. For example, kisses may be totally averted in professional settings and initiated mobsters of the same mafia family might kiss each other.

Even though social rules around kissing vary within and across societies, all kissing “etiquettes” share the common function of regulating and containing the apparent strong intimate connotation of kissing. Kissing is reserved to specific relationships in specific social instances. This implies that kissing is imbued with an underlying universal biological meaning that crosses cultures, hinting at an evolutionary basis older than the cultural conventions themselves.

## 2 TERMS OF ENDEARMENT: SUMMARY OF PREVIOUS HYPOTHESIS

Different hypotheses have been forwarded based on human biology and behavior, but none is consistent with the putative evolution of great ape social behavior in the wild. Some hypotheses propose that women's lips have evolved as a sexual ornament or that kissing is a derived form of “sniffing” for social inspection,^2^ but these hypotheses struggle to explain why kissing takes the form it does.

More promising hypotheses suggest that kissing inherits its form from baby nursing or premastication,^2^ behaviors that, similarly to kissing, involve lip protrusion and/or suck(l)ing movement. However, these hypotheses struggle to explain the context and function of kissing. Kissing does not have an alimentary purpose, nor it happens only by or to infants. A mother or caretaker passing chewed food protrudes its lips, but it shows the opposite of any ingressive or suction movement. Any makeover of the behavior into adulthood and over evolutionary time becomes necessarily speculative.

All nonhuman mammals nurse, and while the mouth plays important tactile functions in most, kissing between social peers across ages and classes has evolved in none. On the other hand, premastication is widely present in primate and bird species with high parental investment, suggesting that the behavior is ancient. However, human cultures still show premastication, particularly among hunter‐gatherers, demonstrating that the behavior has been conserved in the human lineage other than having evolved into an altered set of movements, context, and function. The question of why premastication would have shifted to take on the exact form and function seen today in kissing would, once again, be left answered.

Recent historical studies maintain that great apes show mouth‐to‐mouth kissing in sexual contexts, seemingly offering obvious continuity with humans,^1^ but these claims are based on similes and romanticized descriptions,^2^, ^3^ not scientific studies, similarly to how one might say that a dog “kisses” its owner with a lick on the face.

## 3 KISS KISS BANG BANG: CURRENT EMPIRICAL LIMITATIONS

The evolution of kissing is best understood through the biology and behavior of great apes, who offer living proxies of human's hominid ancestors. Field primatologists are, however, not completely immune from anthropomorphic projections. Mouth–mouth contact in great apes is rare, occurring fundamentally as a behavior of postconflict reconciliation and consolation, often followed by grooming.^4^, ^5^, ^6^ These moments of snout‐to‐snout touch are coined “kissing” as shorthand, in large part due to the quick likeness of the behavior to one that human observers readily recognize for themselves. Whether postconflict “kissing” really involves protruded lips and/or suction is unclear and deserves detailed description and further research. Kissing has also been described as a greeting behavior in chimpanzees,^7^ but alas, without sufficient detail to allow determining the degree to which it mirrors the behavior in humans.

Thus far, a major challenge for assessing hypotheses on the origins of kissing and their evolutionary likelihood is the fact most have overlooked or failed to describe the exact form of the behavior.^1^ Admittedly, how to give a kiss is self‐explanatory for humans, who have first‐hand experience of what a kiss is. However, without the contemplation of the basic, distinctive features of kissing—protruded lips and suction movement—existing hypotheses fail to account why it evolved into its current form as opposed to any another. For example, capuchin monkeys demonstrate and strengthen social bonding by sticking each other's fingers in the eyes and nostrils of friends and kin.^8^ This shows that, while social bonding is undeniably important in primates, it must be accomplished through stipulated forms to be meaningful to others. For a capuchin monkey, perhaps sticking a finger in another's ear is uncanny to peers and of little social benefit, similarly to how a kiss without protruded lips and suction movement may be misintented by the kisser or raise wariness in the receiver about the kiss' intention and genuity.

The focus of most hypotheses has also centered primarily on the sexual context and erotic connotations of mouth‐to‐mouth kissing.^1^, ^2^ From an evolutionary point of view, this implies that general kissing evolved from mouth‐to‐mouth kissing, however, the latter is but a specific case of the former (as above described in Romans, but also in other ancient^1^ and modern societies). The most likely and straightforward evolutionary explanation is that mouth‐to‐mouth kissing evolved from an earlier form of kissing involving the mouth and other body parts.

Finding a lead on the evolutionary origins of kissing requires the use of the term in human *sensu stricto* and the identification of a behavior in great apes that matches its form (lip protrusion + suction movement), context (between individuals of various ages and sexes), and function (bonding between individuals with close affection or kinship) to the greatest extent possible. If such behavior exists in the great ape repertoire, it would be an indication of homology, drawing a putative evolutionary scenario with few gaps to fill as to how, to whom, and when humans kiss the way we do.

## 4 GROOMER'S FINAL KISS

Among terrestrial nonhuman primates, including great apes, the dominant and most prevalent signal of social bonding is grooming.^9^ Grooming consists of picking through the fur/hair of others to remove parasites, dead skin, and debris. Grooming helps to establish and maintain alliances, hierarchies, and group cohesion through social touch, with the consequent release of endorphins, which reduces stress and promotes feelings of well‐being between groomer and groomed, further cementing social ties.^10^ Grooming represents, thus, an ancestral hygienic and affiliative behavior among primates, one that is performed between other‐ and same‐sex and ages, first and foremost between individuals with strong social ties and close kinship.

In comparison with a typical primate, humans groom 89% less than the expected, especially for hygienic purposes.^11^ This is consistent with the loss of fur during human evolution.

Evolutionarily, this means that, as the utilitarian need for grooming decreased among human ancestors, its downstream social functions would have been lost alongside. Some scholars argue that this function was replaced by “vocal grooming,” with vocal exchanges taking over the social functions previously met by grooming, eventually leading to the evolution of human speech and language.^12^ This scenario remains debated, with different strands of research agreeing^13^, ^14^ and disagreeing with the hypothesis.^11^, ^15^

The common notion across schools of thought is, however, that the social function of grooming was *completely* lost and replaced by a disparate suit of behaviors. It has never been considered, nor can it be theoretically excluded, that some vestigial form of grooming persisted, retaining (at least some of) the ancestral social functions.

Grooming wouldn't only have decreased in frequency along the human lineage, it would have also decreased in duration; as human ancestors gradually lost fur, grooming bouts became gradually shorter. That is, less time was necessary to comb (typically with the hands or hands and lips) through a peer's or partner's coat to find debris or a parasite. A grooming bout would have been consummated, however, in the same way—with the groomer touching the groomed with protruding lips and sucking action to latch on and remove a parasite or debris. Regardless of how shorter grooming bouts would have become, they would have preserved this final step. Presumably, up until the ultimate point when two individuals simply performed the last step of grooming, latching on their lips to the other's skin but having discarded the hygienic (and by now obsolete) function of grooming.

This final step—when the groomer closes the grooming bout by kissing the groomed—exhibits parallels in form, context, and function to human kissing to an extent that no other proposed behavior thus far has; Groomer's final kiss (*sensu stricto*) shows protruding lips and suction movement, is performed between various individuals of a community (e.g., not just by or to babies or sexual partners), and among them, those with closer social ties and/or kinship.

A potential means to start gathering support for this hypothesis could involve comparing grooming behavior between populations exhibiting different coat thickness. For example, in captivity, over(allo‐ and/or ‐self)‐grooming among chimpanzees is common, often resulting in conspicuous bands of exposed skin with minimal or no fur. If the “groomer's final kiss hypothesis” is correct, then the prediction is that (while controlling for relevant socioecological factors that may affect the rates of affiliate behaviors), grooming bouts should be expected to relatively shorter in duration in the populations with less fur or thinner coats, and relatively longer in the populations where individuals have thinker coats, and thus, require more time to comb and clean. This will allow confirming that a grooming bout is closed by a groomer's kiss, regardless of its duration.

## 5 QUISS PRO QUO

According to the “groomer's final kiss hypothesis,” it is predicted that mutual, mouth‐to‐mouth kissing emerged in, and stemmed from, social contexts when ancestral apes originally mutually groomed each other at the same time, although this type of grooming is rare among extant great apes compared to one‐way grooming. The establishment of a mouth's kiss as a social convention between intimate partners would presume, however, that mouth‐to‐skin had already become per se formalized. Because the lips and mouth represent one of the parts of the human body with the highest sensitivity to touch, mouth‐to‐mouth kissing is likely to have been driven and maintained also in part for its additional hedonic effects.

## 6 A KISS FOR THE ROAD: CONCLUDING REMARKS

Current comparative evidence supports that kissing isn't a derived signal of affection in humans, it instead represents a surviving devolved, vestigial form of primate grooming that conserved its ancestral form, context, and function. What was once a time‐ and labor‐intensive ritual to cement and strengthen close social ties became gradually compressed until a groomer's final kiss turned into a crystalized symbol of trust and affiliation.

In the wider chronology of human evolution, this hypothesis allows to pin the origins of kissing to a particular point in time in relation to other major behavioral and ecological transformations. For example, it is currently assumed that consonants derive from an ancestral hominid who had a predominant arboreal lifestyle,^16^ pushing the origins of language back in time and up the canopy. Grooming is, however, a typical behavior of terrestrial primates because living at ground level increases parasitic load. As a grooming‐derived behavior, kissing could only have become established once human ancestors started spending a considerable amount of time on the ground, a change that happened only after paleo‐climate change shifted hominid ecology from forested habitats toward drier and more open landscapes.^17^ It is also possible that human ancestors were predisposed to retain the final kiss in a grooming bout due to an overall increased reliance on lip action for the production of communication signals.^18^ For future evolutionary insight into the evolution of human kissing, and other behaviors uniquely exhibited by our species, it will be important to retain in mind and ponder the influence of the broader socioecological, cognitive, and communicative context of human ancestors.

## CONFLICT OF INTEREST STATEMENT

The author declares no conflict of interest.

## Data Availability

The authors have nothing to report.
